# Structural Insights Into the Nuclear Import of Marek’s Disease Virus Large Tegument Protein

**DOI:** 10.1155/av/8716375

**Published:** 2026-06-21

**Authors:** Babu Kanti Nath, Renate H. M. Schwab, Camilla M. Donnelly, Daryl Ariawan, Ole Tietz, Jade K. Forwood, Subir Sarker

**Affiliations:** ^1^ Biosecurity, Gulbali Institute, Charles Sturt University, Wagga Wagga, New South Wales, Australia, csu.edu.au; ^2^ Training Hub Promoting Regional Industry and Innovation in Virology and Epidemiology, Gulbali Institute, Charles Sturt University, Wagga Wagga, New South Wales, Australia, csu.edu.au; ^3^ Dementia Research Centre, Macquarie Medical School, Faculty of Medicine, Health and Human Sciences, Macquarie University, North Ryde, New South Wales, Australia, mq.edu.au; ^4^ Biomedical Sciences & Molecular Biology, College of Medicine and Dentistry, James Cook University, Townsville, Queensland, Australia, health.qld.gov.au; ^5^ Australian Institute of Tropical Health and Medicine, James Cook University, Townsville, Queensland, Australia, health.qld.gov.au; ^6^ Department of Microbiology, Anatomy, Physiology and Pharmacology, School of Agriculture, Biomedicine and Environment, La Trobe University, Melbourne, Victoria, Australia, latrobe.edu.au

**Keywords:** crystallography, EMSA, gallid alphaherpesvirus 2, importins, nuclear trafficking

## Abstract

Marek’s disease (MD) is a highly contagious neoplastic disorder of poultry caused by MD virus (MDV; gallid alphaherpesvirus 2 [GaAHV2]). Infection results in profound immunosuppression, neurological dysfunction, and the development of malignant T‐cell lymphomas. Continued viral evolution has produced increasingly virulent strains capable of partially or fully evading current vaccines, leaving few options for controlling emerging variants. This highlights the importance of identifying new antiviral targets, particularly those involved in the nuclear trafficking events essential for GaAHV2 replication. The UL36 large tegument protein of alphaherpesviruses contains N‐terminal nuclear localization signals (NLSs) thought to guide capsid transport to the nuclear pore complex. However, the specific mechanism by which GaAHV2 UL36 engages the host nuclear import machinery remains unclear. In this work, we defined the NLS within the N‐terminal region of GaAHV2 UL36 and characterized its interaction with importin proteins. Through high‐resolution crystallography and quantitative binding assays, we pinpointed the residues and structural motifs within UL36 that mediate recognition by importin‐α (IMPα) and compared their affinities across different IMPα isoforms. Our structural and biochemical data show that the predicted N‐terminal NLS of GaAHV2 UL36 is essential for IMPα binding. These findings provide a detailed molecular framework for host–virus interactions during GaAHV2 nuclear entry and offer potential avenues for the development of targeted antiviral strategies.

## 1. Introduction

Marek’s disease (MD) leads to profound immunosuppression, neurological abnormalities, and transmissible lymphoproliferative disorder in poultry [1]. Beyond its direct impact on mortality, MDV infection also contributes to secondary infections, reduced productivity, and elevated morbidity in affected birds. Infected birds shed the virus from their feather follicle epithelia, facilitating the spread of the disease among flock members [2, 3]. The etiological agent of MD is *Mardivirus gallidalpha 2*, also referred to as MD virus type 1 (MDV1). This virus is the prototype species within the genus *Mardivirus*, part of the *Alphaherpesvirinae* subfamily, which also includes related avian herpesviruses, such as *Mardivirus gallidalpha3* (GaAHV3) and *Mardivirus meleagridalpha1* (MeAHV1), commonly known as turkey herpesvirus (HVT) [4]. Although GaAHV3 and MeAHV1 share antigenic similarities with MDV, neither virus is pathogenic in chickens. Based on their pathogenicity and virulence, MDV1 strains are further classified into distinct pathotypes, including mild (m), virulent (v), very virulent (vv), and very virulent plus (vv+) strains [5]. Widespread immunization programs have significantly reduced disease prevalence in the 21st century [6]. However, prolonged vaccination practices and incomplete immunity have driven the emergence of increasingly virulent MDV strains, such as vv + MDV and hypervirulent MDV (HV‐MDV) variants, which have been responsible for recurrent outbreaks of MD in vaccinated poultry populations worldwide [7–11]. Currently, there are no antiviral drugs to treat MDV infection, with disease control primarily dependent on vaccination strategies.

In light of the need for alternative MDV control strategies, examination of conserved viral processes, such as the structural characterization of the UL36 NLS‐importin interface may, in the long term, enable the rational design of selective inhibitors that disrupt virus‐specific interactions without broadly impairing host nuclear transport. Herpesvirus capsids travel through the cytoplasm to the nuclear pore complex (NPC) and undergo structural changes, through mechanisms that remain incompletely understood, to facilitate genome release and transport into the nucleus [12]. This process, which is best characterized for herpes simplex virus type 1 (HSV‐1), allows the transcription of the immediate early genes [13]. The large tegument protein VP1‐2, encoded by the UL36 gene, is essential for virus replication and is highly conserved across the herpesvirus family [14–17]. VP1‐2 is a complex, multifunctional protein that plays key roles in various stages of the viral life cycle, such as viral entry, capsid trafficking, and the assembly of new virions [14, 18, 19]. Previous studies on HSV‐1 VP1‐2 have revealed the presence of a functional NLS positioned near the N‐terminal ubiquitin‐specific protease (USP) domain (position _400_GLPKRRRPTWT PPSSVEDLTS_420_) [19–21]. Deletion of this NLS was shown to block capsid attachment to NPCs and prevent the initiation of viral gene expression, while having no effect on virion assembly or extracellular release [19]. This finding suggests that the nuclear targeting of VP1‐2 is essential during the early phases of the herpesvirus life cycle [19]. Given that this NLS is positionally conserved among various herpesvirus orthologues, it is thought to serve a similarly important function during the initial stages of infection across the herpesvirus family [21].

The transportation of proteins into the nucleus is often a tightly regulated process. Among the known nuclear import systems, the IMPα/β1 pathway is the most extensively characterized for mediating protein transport into the nucleus. In this process, importin‐alpha (karyopherin‐alpha, IMPα) binds to a cargo protein carrying a classical nuclear localization signal (cNLS). The resulting cargo‐IMPα complex then associates with importin‐beta‐1 (karyopherin‐beta‐1, IMPβ1) via the N‐terminal importin‐beta‐binding (IBB) domain. This trimeric assembly is subsequently translocated through the NPC and into the nucleus, facilitated by interactions mediated by IMPβ1 and phenylalanine‐glycine (FG) repeat motifs within nucleoporins that line the pore [22, 23]. Once inside the nucleus, RanGTP binds to IMPβ1, causing the dissociation of the cargo–importin complex. Following this, both IMPα and IMPβ1 are transported back to the cytoplasm, where they can participate in additional cycles of nuclear import [24–26]. The number of IMPα isoforms varies across animal species. Yeast possesses a single isoform, while Drosophila and nematodes have three, humans have seven, and six isoforms have been described in poultry [27]. IMPα isoforms are divided into three distinct subfamilies according to their sequence homology. In humans, for instance, the α1 subfamily includes IMPα5 (KPNA1), α6 (KPNA5), and α7 (KPNA6); the α2 subfamily comprises IMPα1 (KPNA2) and α8 (KPNA7), while the α3 subfamily consists of IMPα3 (KPNA4) and α4 (KPNA3) [28, 29]. The central region of IMPα mediates cargo recognition and consists of 10 armadillo (Arm) repeat motifs, each about 42‐43 amino acids long and rich in hydrophobic residues. Proteins bearing a cNLS interact with two specific areas within these Arm repeats: the major binding site, found at Arm repeats 2‐4, and the minor binding site, located at Arm repeats 6–8. The key binding residues within the cNLS engage defined binding pockets on IMPα, with positions P1–P5 corresponding to the major site and P1′–P4′ to the minor site. Typically, monopartite cNLSs, such as that of the SV40 large T‐antigen, bind only to the major binding site, while bipartite cNLSs, such as that described for nucleoplasmin, interact with both the major and minor binding regions [30, 31].

Despite the critical role of nuclear import in herpesvirus replication, the mechanisms by which VP1‐2 proteins from *Alphaherpesvirinae* members infecting animals such as MDV are transported to the nucleus remain poorly understood. In particular, the functional significance of NLSs within these proteins and their interactions with importins (IMPs) have yet to be elucidated. Addressing this gap could reveal novel therapeutic targets for controlling MDV infection. In this study, we investigated the predicted NLS in the large tegument protein of GaAHV2, characterizing its structural and functional features and exploring the molecular basis of its nuclear transport using integrated structural biology and biochemical approaches.

## 2. Materials and Methods

### 2.1. Retrieval and Analysis of Herpesvirus UL36 Gene Sequences

The complete GaAHV2 genome (GenBank accession AF243438.1), along with selected herpesvirus genomes, was retrieved from GenBank. Sequence processing was conducted using Geneious Prime (v2026.1.1). Multiple sequence alignments of the predicted UL36 homologue (UL36 h) were generated in MAFFT (v7.450) using the G‐INS‐i algorithm with a gap‐opening penalty of 1.53 and an offset parameter of 0.123.

### 2.2. Peptide and Gene Construct Design and Synthesis

The UL36 protein sequence of GaAHV2 was used to predict potential NLSs by using cNLS mapper program [32]. Our analysis predicted a putative monopartite NLS with a score of > 5.0 (^467^ARHKRRRPLW^476^). A synthetic peptide was designed based on the predicted NLS amino acid sequence and incorporating a fluorescein isothiocyanate (FITC) label via an aminohexanoic acid (Ahx) linker modification at the N‐terminus. The peptide was synthesized by Macquarie University, Sydney, Australia, via standard fluorenylmethoxycarbonyl (Fmoc) solid‐phase peptide synthesis using a CEM Liberty Blue automated synthesizer (CEM, USA) according to a previously published procedure [33, 34]. Mutant variants of the FITC‐labelled NLS peptides were also designed based on the structural configuration of IMPα binding, incorporating modifications to key residues within the NLS binding interface to assess their impact on IMP recognition. N‐terminally truncated isoforms of IMPα, lacking the autoinhibitory IBB domain, were used in this study, including human IMPα1 (aas 70–529; UniProt: P52292), human IMPα3 (aas 64–521; UniProt: O00629), human IMPα5 (aas 73–538; Uniprot: P52294) and human IMPα7 (aas 73–536; Uniprot: O60684), mouse IMPα2 (aas 70–529; UniProt: P52293). Human isoforms were expressed as fusion proteins with an N‐terminal 6His tag and a tobacco etch virus (TEV) protease cleavage site in the expression vector pET30a(+), as previously described [31, 35]. Mouse IMPα2 was expressed as a fusion protein with N‐terminal 6His tag in pET30a as previously described [31]. Mouse IMPβ1 (Uniprot: P70168) was expressed in pMCSG21 vector with an N‐terminal 6His tag and TEV protease site [36].

### 2.3. Recombinant Expression and Purification

Overexpression of human IMPα1, IMPα3, IMPα5, and IMPα7 and mouse IMPα2 and IMPβ1 was performed in *E. coli* pLysS cells using the autoinduction method [37]. On the other hand, overexpression of both the IMPα1 major and minor mutants was performed in *E. coli* pLysS cells using the autoinduction method [37]. After 32 h of incubation at room temperature, bacterial cultures were harvested by centrifugation at 5232 × g. The resulting cell pellets were resuspended in His buffer A (50 mM phosphate buffer, 300 mM NaCl, 20 mM imidazole, pH 8) at a ratio of 10 mL per 2 L of culture. Cells were lysed through two consecutive freeze‐thaw cycles. To further disrupt the cells, the resuspended pellets were treated with 2 mL of 20 mg/mL lysozyme (Sigma‐Aldrich, USA) and 20 μL of 50 mg/mL DNase (Sigma‐Aldrich, USA) per 50 mL of suspension, incubated at room temperature on a tube roller for 1 h. The lysate was then clarified by centrifugation at 11,269 × g for 40 min. Before purification, the soluble protein fraction was obtained by filtering the clarified lysate through a 0.45 μm low protein‐binding membrane. This filtrate was applied to a 5 mL HisTrap HP column (GE Healthcare, USA) on an ÄKTA pure FPLC system, followed by washing with 20 column volumes of His buffer A. Bound proteins were subsequently released using a stepped imidazole gradient ranging from 20 to 500 mM (ChemSupply, Australia), and the fractions containing the protein of interest were collected and combined. For additional purification, the pooled sample was subjected to size‐exclusion chromatography on a HiLoad 26/600 Superdex 200 column (GE Healthcare, USA) that had been equilibrated in GST buffer A (50 mM Tris, 125 mM NaCl). Fractions eluting at positions consistent with the predicted molecular weights of the IMP proteins were selected, concentrated using 10 kDa Amicon Ultra devices (Merck Millipore, USA), and aliquoted for storage at −80°C. The final purity was examined by SDS–PAGE on a 4%–12% Bis‐Tris Plus gel (Thermo Fisher Scientific) run at 165 V for 30 min, prior to use in downstream experiments.

### 2.4. Crystallization, Data Collection, and Structure Determination

Purified IMPα2 was crystallized using the hanging‐drop vapour‐diffusion method under the known conditions of 0.6 M sodium citrate, 0.1 M HEPES, and 0.01 M DTT (pH 7.0) [38, 39]. Each well contained 300 μL of crystallant solution, and the hanging drops were prepared with 1.5 μL of protein and 1.5 μL of reservoir solution. The IMPα crystals were soaked with FITC‐GaAHV NLS prior to being cryoprotected in reservoir solution plus 20% glycerol, before being flash‐cooled in liquid nitrogen. The crystals were diffracted at the Diamond Light Source on beamline i04 [40, 41]. The data were integrated and scaled using DIALS [42]. Data were merged and scaled using AIMLESS [43], phased by molecular replacement in Phaser MR [44] using PDB 6BVT as a model [45] and then refined using Refmac [46, 47] and Phenix [48] with iterative model building performed in COOT [49]. The protein–protein interactions were calculated by PDBePISA [50]. Coordinates for the GaAHV2 UL36 NLS–IMPα structure were deposited into the Protein Data Bank under the accession code 9OVZ.

### 2.5. Fluorescence Polarization

Synthetic FITC‐labelled peptide (2 nM final concentration) was incubated with two‐fold serial dilutions of IMP isoforms, starting at 20 μM, across 23 wells in a final volume of 200 μL per well in GST buffer A (50 mM Tris, 125 mM NaCl, pH 8.0). Fluorescence polarization measurements were performed using a CLARIOstar Plus plate reader (BMG Labtech, Germany) in 96‐well black Fluotrac microplates (Greiner Bio‐One, Austria), essentially as previously described [39, 51]. Each assay was conducted in triplicate, each containing a negative control (no IMP‐binding partner). Data were analysed with GraphPad Prism (Prism 9, version 9.3.1), and a binding curve was fitted to the one‐site‐specific binding least square fit function (accounts for ligand depletion) to determine the dissociation constant (*K*_*D*_) and maximum binding (Bmax). Bmax data were not provided in the FP graph.

### 2.6. Electromobility Shift Assays

A FITC‐labelled peptide (10 μM) was incubated with 20 μM of each IMPα isoform in a 20 μL reaction containing 3 μL of 50% (v/v) glycerol, with GST buffer A (50 mM Tris, 125 mM NaCl, pH 8.0) making up the remaining volume as previously described [34, 39, 52]. Samples were resolved on a 1% (w/v) agarose gel in TB buffer (45 mM Tris, 45 mM boric acid, pH ∼8.5) at 80 V for 2 h. Gels were imaged using a SYBR Green filter on a Bio‐Rad Gel Doc system to visualize FITC–peptide complexes. Subsequently, gels were stained with Coomassie brilliant blue R‐250, destained overnight (10% ethanol, 10% acetic acid), and reimaged to assess total protein migration and complex formation.

## 3. Results

### 3.1. Genetic Variability of the GaAHV2 UL36 Gene

Evaluation of the full‐length UL36 protein sequences from GaAHV2 isolates showed that the gene is highly conserved, with amino acid identities ranging from 81% to 100%. In contrast, comparison with UL36 homologues from other herpesviruses indicated substantial divergence (Supporting Table S1). The highest pairwise similarity was observed with GaAHV1 (∼50% identity), followed by HVTs (∼47%). Considerable variation in UL36 protein length was also evident among the herpesviruses examined, which may partly explain the reduced sequence similarity across species (Supporting Table S1). Inspection of conserved functional motifs showed that GaAHV2 UL36 lacks a canonical nuclear localization signal (NLS) corresponding to that reported in HSV‐1 (GLPKRRRPTWTPPSSVEDLTS) (Supporting Figure S1). However, intraspecies comparison demonstrated strong conservation across GaAHV2 UL36 sequences. Computational prediction using cNLS Mapper, supported by manual inspection, identified a putative NLS within residues 467–476. This region was completely conserved across all analysed GaAHV2 isolates (Supporting Figure S1).

### 3.2. Biochemical Determination of GaAHV2 NLS Preference for Both IMPα and IMPβ1 Isoforms

**FIGURE 1:**
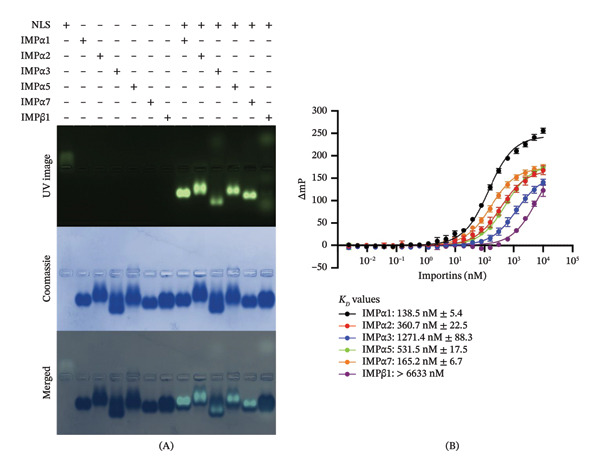
Interaction of GaAHV2 UL36 NLS with various IMPs. (A) EMSA demonstrating the binding of the GaAHV2 UL36 NLS peptide to different IMPα isoforms. The NLS peptides were labelled with FITC and an Ahx linker, allowing visualization under UV light (green fluorescence). Protein bands were detected by Coomassie blue staining (blue). The EMSA data represent results from three independent experiments. (B) FP assay assessing the binding affinity between the GaAHV2 UL36 NLS and the corresponding IMP isoforms. Values are presented as the mean ± standard error (SE) from three separate experiments. Binding constants (*K*_*D*_) were determined according to the procedures outlined in the Materials and Methods section.

To evaluate whether the predicted GaAHV2 NLS binds to host nuclear import receptors IMPα and IMPβ1 and to identify any preference for specific receptor isoforms, we conducted biochemical binding assays. EMSAs were performed to qualitatively assess the interactions between the GaAHV2 NLS and various IMP isoforms, including IMPα family members (human IMPα1, mouse IMPα2, human IMPα3, human IMPα5, and human IMPα7) and mouse IMPβ1. Three independent experiments confirmed that the predicted NLS binds to IMPα isoforms and to IMPβ1with lower affinity. It is evident that there is minimal shifting of IMPs upon binding to the NLS, which is not unexpected in the Coomassie‐stained gels, as IMP is a large protein of approximately 52 kDa. However, the shift of the FITC‐labelled NLS after binding to IMP is clearly visible in the UV image (Figure 1A).

To further assess the interactions and determine the dissociation constants (*K*_*D*_) of the IMP/peptide complexes, quantitative FP assays were performed following established methods [39, 53–57] (Figure 1B). The NLS demonstrated higher binding affinities, interacting with IMPα1 (*K*_*D*_ = 138.5 nM), IMPα7 (*K*_*D*_ = 165.2 nM), IMPα2 (*K*_*D*_ = 360.7 nM), IMPα5 (*K*_*D*_ = 531.5 nM), and IMPα3 (*K*_*D*_ = 1271.4 nM), followed by IMPβ1 (*K*_*D*_ = 6633.2 nM). These findings indicate that GaAHV‐2 NLS binds to different IMPs with varying affinities and supports the notion that GaAHV2 UL36 NLS binds to IMPα to mediate nuclear import. However, low‐affinity binding of IMPβ1 to the NLS peptides of UL36 from GaAHV2 is consistent with noncanonical interactions which is generally attributed to the presence of dispersed basic residues that can weakly engage the HEAT‐repeat surface of IMPβ1 in the absence of IMPα.

### 3.3. GaAHV2 UL36 Binds to IMPα With a Monopartite NLS

**FIGURE 2:**
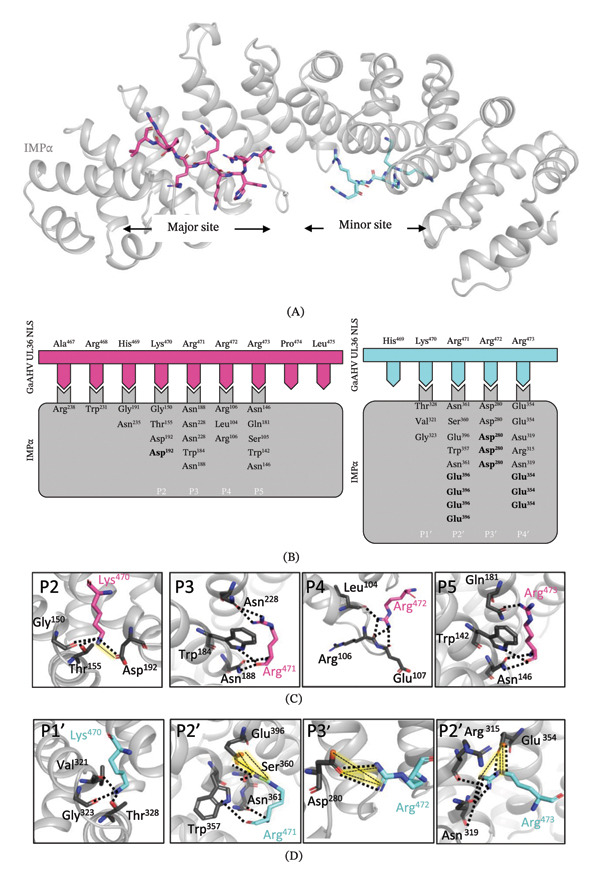
Resolved crystal structure of IMPα bound to GaAHV2 UL36 NLS. (A) The IMPα structure (grey cartoon) displays the GaAHV2 UL36 NLS peptide at the major binding site (pink sticks) and minor binding site (aquamarine sticks). (B) Schematic representation of the interactions between GaAHV UL36 NLS and IMPα, with residues at the major binding site highlighted in pink and those at the minor binding site in aquamarine. Interfacing amino acids from IMPα are indicated by grey boxes, with salt bridges denoted in bold text. Defined cargo binding pockets are labelled in white. (C) Close‐up images of GaAHV UL36 NLS amino acid side chains (pink) and IMPα interfacing residues (grey sticks) with interactions calculated by PDBePISA, showcasing binding pockets P2–P5. (D) Close‐up images of GaAHV2 UL36 NLS amino acid side chains (aquamarine sticks) and IMPα interfacing residues (grey sticks), also with interactions calculated by PDBePISA, highlighting binding pockets P1′–P4′. Molecular image generated in Pymol.

The structure was resolved to 2.4 Å and the resulting model has a single molecule of IMPα and two chains of GaAHV2 UL36 NLS in the asymmetric unit. The diffraction data were indexed to the space group P2_1_2_1_2_1_ with the unit cell parameters *a* = 77.88, *b* = 91.06, and *c* = 99.89 (see Table 1 for detailed statistics). Electron density within the well‐characterized IMPα major binding site accommodates the GaAHV2 UL36 NLS sequence ^467^ARHKRRRPL^475^ with Lys^470^ occupying the P2 site (comprised of Gly^150^, Thr^155^, and Asp^192^). The interaction is mediated by 21 hydrogen bonds and one salt bridge, with significant contributions from Arg^471^ and Arg^473^ in the P3 and P5 binding pockets, respectively (Figure 2) [50]. The positioning of His^469^ side chain was found to be dynamic within the crystal structure, interacting with IMPα Gly^191^ oxygen with a partial occupancy of 0.6 or directed away from the interface with an occupancy of 0.4. Electron density corresponding to a second chain of GaAHV2 UL36 was found to occupy the IMPα minor binding site with residues ^469^HKRRR^473^ forming the interface. This interaction is mediated by 15 hydrogen bonds and 10 salt bridges with the greatest contribution of energy coming from Arg^471^ in the P2′ site, contributing five hydrogen bonds and four salt bridges (Table 2).

**TABLE 1:** Data collection and refinement statistics for the structure of importin‐α2 in complex with GaAHV2 NLS.

GaAHV2 NLS and IMPα2 (PDB code: 9OVZ)	
*Data collection (high-resolution statistics in parentheses)*	
Wavelength	0.95373
Data‐collection temperature (K)	298
Detector type	Eiger2 XE 16M
Detector	Pixel
Resolution range (Å)	29.1–2.4 (2.486–2.4)
Space group	P2_1_2_1_2_1_
Unit cell (Å); (°)	77.88 91.06 199.89; 90 90 90
Total reflections	383774 (36043)
Unique reflections	28404 (2777)
Multiplicity	13.5 (13.0)
Completeness (%)	99.87 (99.82)
Mean I/σ (I)	25.74 (1.85)
Wilson B‐factor Å^2^	54.24
R_pim_	0.01863 (0.228)
CC 1/2	0.997 (0.918)

*Refinement*	
R_work_	0.2106 (0.2513)
R_free_	0.2253 (0.2719)
No. of nonhydrogen atoms	3388
Macromolecules	3
Solvent	39
Protein residues	434
Bond length r.m.s.d (Å)	0.013
Bond angle r.m.s.d (°)	1.49
Ramachandran favoured (%)	97.90
Ramachandran allowed (%)	2.10
Ramachandran outliers (%)	0.00

**TABLE 2:** Hydrogen bond and salt bridge interactions between GaAHV2 NLS and mouse IMPα2 as determined by using PDBePISA.

Mouse IMPα2	GaAHV2 NLS1
*Hydrogen bonds (major site)*	
GLY 191 [O]	HIS 469 [ND1]
GLY 150 [O]	LYS 470 [NZ]
THR 155 [OG1]	LYS 470 [NZ]
ASP 192 [OD1]	LYS 470 [NZ]
ASN 188 [OD1]	ARG 471 [N]
ASN 228 [OD1]	ARG 471 [NH1]
ASN 228 [OD1]	ARG 471 [NH2]
ARG 106 [O]	ARG 472 [NH1]
LEU 104 [O]	ARG 472 [NH1]
ARG 106 [O]	ARG 472 [NH2]
GLU 107 [ O]	ARG 472 [NH2]
ASN 146 [OD1]	ARG 473 [N]
GLN 181 [OE1]	ARG 473 [NH1]
SER 105 [O]	LEU 475 [N]
ARG 238 [NH2]	ALA 467 [O]
TRP 231 [NE1]	ARG 468 [O]
ASN 235 [ND2]	HIS 469 [O]
TRP 184 [NE1]	ARG 471 [O]
ASN 188 [ND2]	ARG 471 [O]
TRP 142 [NE1]	ARG 473 [O]
ASN 146 [ND2]	ARG 473 [O]

*Salt bridges (major site)*	
ASP 192 [OD1]	LYS 470 [NZ]

*Hydrogen bonds (minor site)*	
THR 328 [OG1]	LYS 470 [NZ]
VAL 321 [O]	LYS 470 [NZ]
GLY 323 [O]	LYS 470 [NZ]
ASN 361 [OD1]	ARG 471 [N]
SER 360 [OG]	ARG 471 [NH2]
GLU 396 [OE1]	ARG 471 [NH2]
ASP 280 [OD1]	ARG 472 [NH1]
ASP 280 [OD1]	ARG 472 [NH2]
GLU 354 [OE1]	ARG 473 [NE]
GLU 354 [OE2]	ARG 473 [NE]
ASN 319 [OD1]	ARG 473 [NH1]
ARG 315 [O]	ARG 473 [NH2]
ASN 319 [OD1]	ARG 473 [NH2]
TRP 357 [NE1]	ARG 471 [O]
ASN 361 [ND2]	ARG 471 [O]

*Salt bridges (minor site)*	
GLU 396 [OE1]	ARG 471 [NH1]
GLU 396 [OE2]	ARG 471 [NH1]
GLU 396 [OE1]	ARG 471 [NH2]
GLU 396 [OE2]	ARG 471 [NH2]
ASP 280 [OD1]	ARG 472 [NH1]
ASP 280 [OD1]	ARG 472 [NH2]
ASP 280 [OD2]	ARG 472 [NH2]
GLU 354 [OE1]	ARG 473 [NE]
GLU 354 [OE2]	ARG 473 [NE]
GLU 354 [OE1]	ARG 473 [NH2]

### 3.4. Mutational Studies Confirm Monopartite Nature of GaAHV2 NLS

**FIGURE 3:**
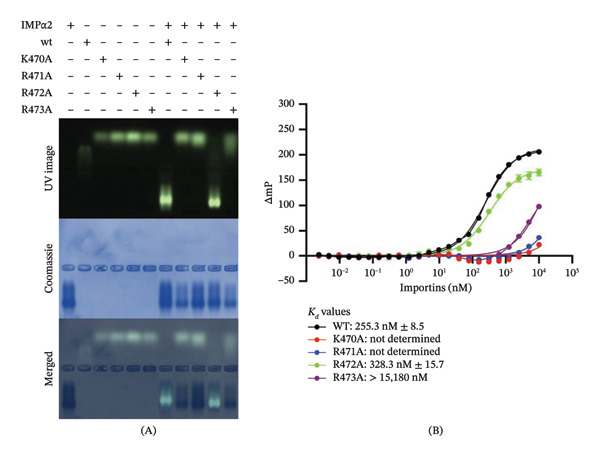
Binding affinities of IMPα2 with GaAHV2 UL36 NLS mutants. (A) EMSA analysis showed no detectable binding between IMPα2 and the NLS mutant peptides, except for the R472A mutant. Each peptide was labeled at the N‐terminus with FITC and an Ahx linker and visualized under UV light (green). Proteins were stained with Coomassie blue, and the overlay images display both the FITC‐tagged peptides and the Coomassie‐stained IMPα proteins. The EMSA results represent data from three independent experiments. (B) Fluorescence polarization assays quantified direct binding interactions between the NLS mutants and the indicated IMPα2 isoform. *K*_*D*_ values are presented as mean ± standard error of the mean (SEM) from three separate experiments. Nonlinear regression analysis for *K*_*D*_ calculation was performed using GraphPad Prism, as detailed in the Materials and Methods section.

The GaAHV2 NLS sequence (^467^ARHKRRRPL^475^) features a continuous cluster of basic residues that engages IMPα2 (Figure 2), leading us to propose that it functions as a monopartite signal. To assess this, we examined how specific residues contribute to IMPα2 recognition using EMSA and fluorescence polarization assays. Alanine substitutions at K470, R471, and R473, positions that align with the P2, P3, and P5 pockets of the IMPα2 major binding cleft, greatly diminished peptide and IMPα2 complex formation in EMSA (Figure 3A). Consistent with these observations, the same mutations produced marked increases in *K*_*D*_ values in FP assays (Figure 3B), reflecting reduced binding strength. Overall, these data highlight the importance of residues 470 to 473 in establishing high affinity interactions with IMPα2, supporting the conclusion that this region constitutes a functional monopartite NLS in GaAHV2.

**FIGURE 4:**
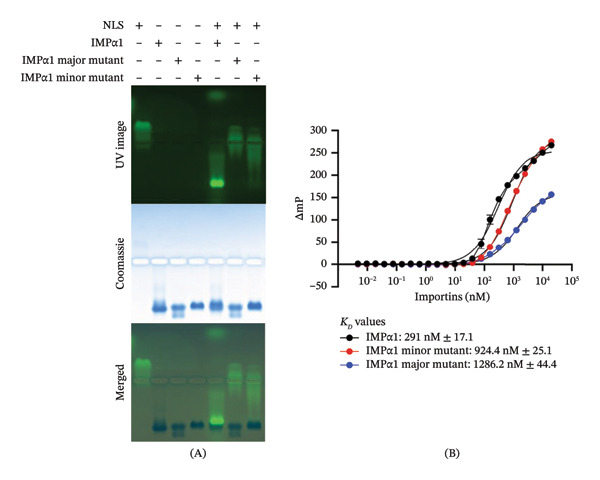
Interaction analysis of IMPα1 major and minor site mutants with GaAHV2 UL36 NLS. (A) EMSA indicates a lack of binding between the GaAHV2 large tegument protein NLS and the IMPα1 mutants at both the major and minor binding sites. The GaAHV2 UL36 NLS peptides were labeled at the N‐terminus with FITC and included an Ahx linker, allowing visualization under UV light (green fluorescence). Proteins were visualized using Coomassie blue staining (red). The EMSA results are representative of three separate experiments. (B) FP assay quantifying the binding affinity of GaAHV2 UL36 NLS to the IMPα1 major and minor site mutants. Data represent the mean ± standard error of the mean (SEM) from three independent experiments. Dissociation constants (*K*_*D*_) were calculated by nonlinear regression analysis using GraphPad Prism, following the methods described in the Materials and Methods section.

To further validate the binding mode of the GaAHV2 NLS, we assessed its interaction with ΔIBBIMPα1 mutants, carrying point substitutions in either the major or minor binding sites, using EMSA (Figure 4A) and FP assays (Figure 4B). Mutation of Asp^192^ to Lys (D^192^K) within the major binding site of IMPα1 led to a marked reduction in comigration with the GaAHV2 NLS in EMSA (Figure 4A) and significantly impaired binding affinity, as evidenced by > 4‐fold increase in *K*_*D*_ value in FP analysis (Figure 4B). In the same way, substitution of Glu^396^ with Arg (E^396^R) in the minor binding site also reduced comigration in EMSA (Figure 4A) and caused a moderate decrease in binding affinity relative to the wild‐type protein (Figure 4B). These findings indicate that while the GaAHV2 NLS primarily interacts with the major binding site of IMPα1, secondary contacts at the minor site may also contribute to stabilizing the NLS–IMP interaction.

## 4. Discussion

Herpesviruses are DNA viruses that replicate within the cell nucleus of host cells, a process that requires understanding viral protein behaviour and localization to develop effective antiviral strategies [58, 59]. Research on HSV‐1 showed that it encodes 21 proteins localized in the cytoplasm or subcytoplasmic membranes, 16 proteins confined to the nucleus or subnuclear regions, and several others distributed between both the cytoplasm and the nucleus [60]. In addition, the majority of herpesvirus envelope proteins are typically found in the cytoplasm, whereas most capsid proteins are predominantly or exclusively localized within the nucleus. This pattern of subcellular distribution implies that the localization of each protein is closely linked to its role in the viral replication process [60]. VP1‐2 is a large, conserved structural protein essential for multiple stages of herpesvirus replication, including genome transport to the nucleus following entry [19]. Previous findings demonstrate that while the VP1‐2 NLS is not required for virion morphogenesis, it is essential for the efficient targeting of incoming capsids to the nuclear pore, a critical prerequisite for initiating productive infection. Given the high conservation of this motif across herpesviruses, it is likely that this mechanism is broadly shared among members of the Herpesviridae family [19, 21]. Other findings suggest that UL36‐encoded USP of MD virus is essential for viral replication, with its N‐terminal region and a conserved segment encompassing the catalytic cysteine (C98) being indispensable, and accumulating evidence suggests that USP contributes to the structural integrity and stability of pUL36, thereby influencing replication and oncogenesis beyond its enzymatic activity [61]. Given that nuclear import of UL36 depends on recognition of its NLS by IMP receptors, the demonstrated requirement for USP‐mediated structural integrity raises the possibility that this domain indirectly regulates nuclear trafficking by maintaining a conformation competent for IMP binding.

The UL36 gene of GaAHV2 exhibits strong intraspecies conservation (81%–100% amino acid identity). In contrast, the relatively low similarity with other herpesviruses (∼50% with GaAHV1 and ∼47% with HVTs) indicates substantial evolutionary divergence, likely influenced by differences in protein length and lineage‐specific structural variation. The absence of a canonical HSV1–like NLS motif suggests variation in nuclear targeting mechanisms; however, the identification of a fully conserved putative NLS within residues 467–476 across all isolates supports its functional relevance. Collectively, these findings highlight the evolutionary conservation of UL36 within GaAHV2 alongside divergence from related viruses and suggest that the conserved NLS motif may play a critical role in viral protein trafficking, warranting further experimental validation.

HSV‐1 utilizes multiple nuclear localization mechanisms during infection [62–64]. For example, HSV‐1 relies on the IMPα/β1 pathway to ensure the nuclear localization of its DNA polymerase processivity factor UL42 [65], whereas the tegument viral protein VP16 of HSV‐1 interacts with the host cell factor HCF‐1 for nuclear localization [66]. However, the exact mechanism of nuclear transport in GaAHV2 has remained unclear. To explore this, we analysed the classical NLS region in the large tegument protein of GaAHV2, employing structural and biophysical approaches to understand its interactions with cellular IMPs. We characterized the UL36–IMPα2 complex and identified a unique monopartite NLS in GaAHV2. Biochemical analysis demonstrated binding of FITC‐labelled NLS peptide with all tested IMPα isoforms. The predicted NLS interacted with IMPα1 with higher affinity, followed by IMPα7 and IMPα2 (Figure 1B). These findings suggest that GaAHV2 large tegument protein can bind to different IMPαs through NLS within this basic cluster region, supporting its ability to enter the nucleus via classical nuclear import pathways. However, the observed low‐affinity binding of IMPβ1 to the NLS peptides of pUL36 from GaAHV2 is consistent with noncanonical interactions. These NLS regions are enriched in basic residues but do not fully conform to a high‐affinity IMPβ1‐specific binding motif, which likely explains the weaker binding compared to IMPα. We propose that this interaction reflects a transient or opportunistic association rather than a primary import pathway. In the context of infected cells, such low‐affinity interactions could still contribute to import efficiency under conditions of high local protein concentration or limited availability of IMPα. In the classical import pathway, proteins bearing a NLS are identified by IMPα, which subsequently forms a complex with IMPβ1 to facilitate their transport through the NPC. In contrast, unconventional pathways bypass the NLS and IMPα/β1 machinery, utilizing alternative mechanisms such as passive diffusion, direct binding to nucleoporin protein at the NPC, and IMPβ1‐dependent pathways for nuclear entry [67, 68]. To explore these possibilities in greater detail, high‐resolution crystal structures of the interaction interface between IMPα2 and GaAHV2 NLS were determined.

GaAHV2 UL36 binds to IMPα bound at the major and minor binding sites as a classical monopartite NLS. The observed binding at the minor site is likely attributed to an artefact of crystallization, specifically oversaturation of the major site. Structural analysis of the GaAHV2 UL36 NLS in complex with IMPα2 revealed a highly specific and extensive interaction within the well‐characterized major binding site of IMPα2. The electron density clearly accommodated the UL36 NLS sequence ^467^ARHKRRRPL^475^, with Lys^470^ positioned within the P2 pocket, formed by Gly^150^, Thr^155^, and Asp^192^. This interaction was stabilized by a network of 21 hydrogen bonds and one salt bridge, with Arg^471^ and Arg^473^ contributing significantly to binding affinity through interactions within the P3 and P5 pockets, respectively. Notably, the His^469^ side chain exhibited dynamic positioning, alternating between interacting with IMPα2 Gly^191^ oxygen with a partial occupancy and adopting an alternate conformation directed away from the interface. This binding conformation, in particular the Lys within the P2 binding pocket, exemplifies a classical monopartite NLS, which has been well characterized for numerous viral and host proteins [69].

In alphaherpesviruses, the NLS is located within a less conserved linker region, with the exception of a central basic region of approximately 40 residues, the longest among VP1‐2 homologues. This region consists of a conserved N‐terminal basic cluster (R4), a less conserved C‐terminal basic cluster (R5), and an intervening proline/serine/threonine‐rich linker, which may regulate NLS functionality under specific conditions [21]. Bipartite NLS motifs generally interact with IMPα, which contains both major and minor binding pockets for NLS recognition [70]. The linker region connecting the upstream and downstream basic clusters is capable of interacting with IMPα, which may influence the binding process [21]. Interestingly, a similar bipartite arrangement is also found in the HSV‐1 DNA polymerase catalytic subunit UL30 and its processivity factor UL42, both of which possess bipartite NLS motifs, a notable difference from the monopartite NLS motifs found in β‐ and γ‐herpesviruses [65, 71–74].

VP1‐2 or UL36 homologues of β‐ and γ‐herpesviruses exhibit a conserved organization, characterized by a basic region positioned between the USP domain and the main protein body. Within this conserved framework, however, distinct family‐specific variations are evident, suggesting virus‐specific adaptations. Notably, NLS motifs from HSV, VZV, HCMV, EBV, and HHV‐8 have each been shown to function as strong NLSs, either in β‐galactosidase assays or when substituted into HSV VP1‐2 [21]. Earlier studies demonstrated that the NLS in VZV operates strictly as a bipartite motif, whereas in HSV, it can act in either monopartite or bipartite form. For instance, HSV R4 alone is sufficient to drive nuclear import in protein transfer assays; however, deletion or mutation of the adjacent R5 markedly reduces efficiency, highlighting the bipartite requirement for optimal activity. A striking example is the substitution of Lys^428^ with alanine, which almost completely abolished nuclear import and viral replication, emphasizing the indispensable role of this residue despite the presence of multiple basic residues nearby [21]. In contrast, our findings reveal a single basic cluster within UL36 of GaAHV2 that functions as a classical monopartite NLS. Structural analysis of the GaAHV2 NLS in complex with IMPα2 demonstrates that it conforms to the canonical monopartite consensus, K[K/R]x[K/R], and a very strong requirement for the first position (bold). This structural evidence provides the first direct characterization of a monopartite NLS in UL36 of GaAHV2 and aligns with previous reports underscoring the essential requirement for a lysine at the first position [20].

The EMSA analysis of NLS mutations targeting residues involved in binding to IMPα′s major site revealed that substitutions at K^470^, R^471^, and R^473^, key residues interacting with the P2, P3, and P5 pockets of IMPα2, resulted in weakened binding. This was evidenced by diminished peptide comigration in EMSA and elevated *K*_*D*_ values measured in fluorescence polarization assays (Figure 3). These findings underscore the crucial role of residues 470–473 in mediating strong interactions with IMPα2 and emphasize their importance in the efficient recognition of the GaAHV2 NLS. Additionally, mutation of a critical residue within IMPα1’s major binding pocket (Asp^192^ to Lys, D^192^K) led to a marked reduction in NLS comigration during EMSA (Figure 4A) and a significant decline in binding affinity, as indicated by increased dissociation constants in FP assays (Figure 4B). Mutation of Glu^396^ to Arg (E^396^R) in the minor binding pocket of IMPα1 also decreased binding and comigration, although to a lesser extent than the major site mutation (Figure 4A, B). These observations indicate that the GaHV2 NLS interacts with both the major and minor binding sites of IMPα1, with a stronger dependence on the major site, as reflected by the greater effect of the D^192^K substitution on binding affinity. These results align with previous studies showing that detergent‐extracted HSV virions can associate with nuclear pores in vitro, a process partially inhibited by antibodies against nucleoporins or IMPβ [62]. Although IMPβ has been shown to facilitate capsid binding to isolated nuclei, specific viral receptors involved in this interaction remain unidentified [62].

First limitation of this study is the inability to perform binding assays using the full‐length UL36 h protein, primarily due to its large size, the technical difficulties associated with fluorescent labeling, and challenges in recombinant expression. To address this, we utilized a fluorescently labeled synthetic peptide representing the predicted NLS region. This peptide specifically interacted with nuclear import receptors, providing functional evidence supporting its role in nuclear import. Future studies using truncated or full‐length constructs will be important to further define the mechanistic details of UL36h–IMP interactions. Second limitation of this study is the absence of functional validation in the context of viral infection. While our biochemical data demonstrate that the UL36 NLS interacts with IMPs, the impact of this interaction on nuclear entry, viral replication, and spread remains to be determined. Moreover, given that UL36 is a large multifunctional tegument protein, mutation of its NLS may produce pleiotropic effects that complicate interpretation of viral phenotypes. Therefore, careful design and characterization of UL36 NLS mutant viruses will be essential to directly assess its role during infection, and this represents an important direction for future work. Third limitation arises from our use of human and mouse IMPα isoforms rather than proteins derived from chicken, the natural host. The specificity of NLS sequences for various IMP isoforms is not fully characterized and remains poorly understood. However, structural studies suggest that NLS binding affinities are generally conserved across IMPα isoforms, as their NLS‐binding grooves show a high degree of conservation [45, 75–77]. It has been proposed that specificity may be influenced by the linker region in bipartite NLSs [31] or by differences in the ARM repeats outside the conserved binding pockets [45]. While the amino acid sequences of human and chicken IMPα isoforms differ, they share sequence identities ranging from 82% to 99%, specifically α1 (82%), α3 (99%), α4 (98%), α5 (95%), α6 (94%), and α7 (94%) [78], our analysis showed that major and minor binding sites of chicken and human IMPα are highly conserved (Supporting Figure S2). Likewise, the amino acid sequence identity between mouse IMPβ1 and chicken IMPβ1 is about 97.37% (Supporting Figure S2).

To assess the conservation of NLS recognition between chicken (*Gallus gallus*) and mouse IMPs, residues 70–528 of chicken IMPα1 (NCBI accession NP_001006209.2) were modeled using AlphaFold 3. Structural comparison revealed strong similarity between the modeled chicken IMPα1 and the crystal structure of mouse IMPα2 (Supporting Figure S3). Superimposition showed that both proteins maintain the characteristic ARM repeat fold typical of IMPα family members (Supporting Figure S3B). Notably, the modelled chicken IMPα1 aligned closely with mouse IMPα2 at the major NLS‐binding site where the GaAHV2 NLS peptide was docked. Examination of the binding interface indicated that key residues involved in NLS recognition in mouse IMPα2 are strictly conserved in chicken IMPα1 (Supporting Figure S3B). These conserved residues within the major binding pocket engage in equivalent interactions with the NLS peptide, supporting functional conservation of NLS recognition across these species. Furthermore, previous studies have successfully employed human and mouse IMPα proteins to investigate viral NLSs from frog and psittacine adenoviruses as well as abalone herpesvirus [34, 39, 52, 56], lending further credibility to this approach. Nevertheless, future investigations using chicken IMPα will be important to validate host‐specific interactions and to enhance the biological relevance of our findings.

## 5. Conclusions

Our results suggest that the predicted N‐terminal NLS of the GaAHV2 large tegument protein facilitates nuclear import via the IMPα/β pathway, although other transport mechanisms may also play a role. It is probable that herpesvirus nuclear localization involves species‐specific and multiple transport routes. To gain a comprehensive understanding of this process, further studies incorporating subcellular localization assays and investigation of alternative nuclear import pathways are warranted. While this work centred on the GaAHV2 large tegument protein, examining whether similar nuclear transport mechanisms operate in other gallid alphaherpesviruses through comparative analysis of their large tegument proteins would be informative. Together, these insights enhance our knowledge of GaAHV2 biology and may support the development of selective inhibitors that specifically target virus–host interaction interfaces while minimizing disruption of essential host nuclear transport processes, thereby contributing broadly to herpesvirus research.

## Author Contributions

Babu Kanti Nath: writing–review and editing, methodology, investigation, data curation, software, formal analysis, visualization, and writing–original draft preparation. Renate H. M. Schwab: writing–review and editing, software, data curation, formal analysis, and visualization. Camilla M. Donnelly: writing–review and editing, investigation, data curation, software, formal analysis, visualization, and writing–original draft preparation. Daryl Ariawan and Ole Tietz: methodology, resources, and writing–review and editing. Jade K. Forwood and Subir Sarker: conceptualization, funding acquisition, project administration, resources, validation, and writing–review and editing.

## Funding

Subir Sarker was supported by an Australian Research Council Discovery Early Career Researcher Award (DE200100367), funded by the Australian Government.

## Disclosure

The funding body had no involvement in the study design, data acquisition or analysis, the decision to publish, or the preparation of this manuscript.

## Ethics Statement

The authors have nothing to report.

## Conflicts of Interest

The authors declare no conflicts of interest.

## Supporting Information

Additional supporting information can be found online in the Supporting Information section.

## Supporting information


**Supporting Information** Supporting Figure S1: comparative analysis of the UL36 R1 region and nuclear localization signal (NLS) across selected herpesviruses. Supporting Figure S2: alignment of human/mouse and chicken IMP amino acid sequences. Supporting Figure S3: conservation of the IMP⍺ major binding site between mouse IMP⍺1 (named as IMP⍺2 in our study) and chicken IMP⍺1. Residues 70–528 of chicken (*Gallus gallus*) IMP⍺1 (GenBank accession no. NP_001006209.2) were modelled using AlphaFold 3 (494**–**528 hidden in figure). Supporting Table S1: amino acid sequence identities among selected herpesvirus UL36 genes.

## Data Availability

The data that support the findings of this study are openly available in Protein Data Bank at https://www.rcsb.org/, reference number 9OVZ.
