# Supplementary material for: Structural Insights Into the Nuclear Import of Marek’s Disease Virus Large Tegument Protein
Source: Adv Virol. 2026 Jun 21;2026:8716375. doi: 10.1155/av/8716375 (PMC13284491; doi:10.1155/av/8716375)
Supplement: Supplementary file 1 — Supporting Information Supporting Figure S1: comparative analysis of the UL36 R1 region and nuclear localization signal (NLS) across selected herpesviruses. Supporting Figure S2: alignment of human/mouse and chicken IMP amino acid sequences. Supporting Figure S3: conservation of the IMP⍺ major binding site between mouse IMP⍺1 (named as IMP⍺2 in our study) and chicken IMP⍺1. Residues 70–528 of chicken (Gallus gallus) IMP⍺1 (GenBank accession no. NP_001006209.2) were modelled using AlphaFold 3 (494–528 hidden in figure). Supporting Table S1: amino acid sequence identities among selected herpesvirus UL36 genes. [file AV-2026-8716375-s001.pdf]

Supplementary File

Structural insights into the nuclear import of Marek's disease virus large tegument protein

Babu Kanti Nath[1*], Renate H. M. Schwab[1], Camilla M. Donnelly[2], Daryl Ariawan[3,] Ole Tietz[3], Jade K. Forwood[1,2*] and Subir Sarker[4,5,6*]

[1]Biosecurity, Gulbali Institute, Charles Sturt University, Wagga Wagga, New South Wales, Australia.
[2]Training Hub Promoting Regional Industry and Innovation in Virology and Epidemiology, Gulbali Institute, Charles Sturt University, Wagga Wagga, New South Wales, Australia.
[3]Dementia Research Centre, Macquarie Medical School, Faculty of Medicine, Health and Human Sciences, Macquarie University, North Ryde, New South Wales, Australia.
[4]Biomedical Sciences & Molecular Biology, College of Medicine and Dentistry, James Cook University, Townsville, Queensland, Australia.
[5]Australian Institute of Tropical Health and Medicine, James Cook University, Townsville, Queensland, Australia.
[6]Department of Microbiology, Anatomy, Physiology and Pharmacology, School of Agriculture, Biomedicine and Environment, La Trobe University, Melbourne, Victoria, Australia.

* = Correspondence: bnath@csu.edu.au (B. K. Nath), jforwood@csu.edu.au (J. K. Forwood), subir.sarker@jcu.edu.au (S. Sarker).

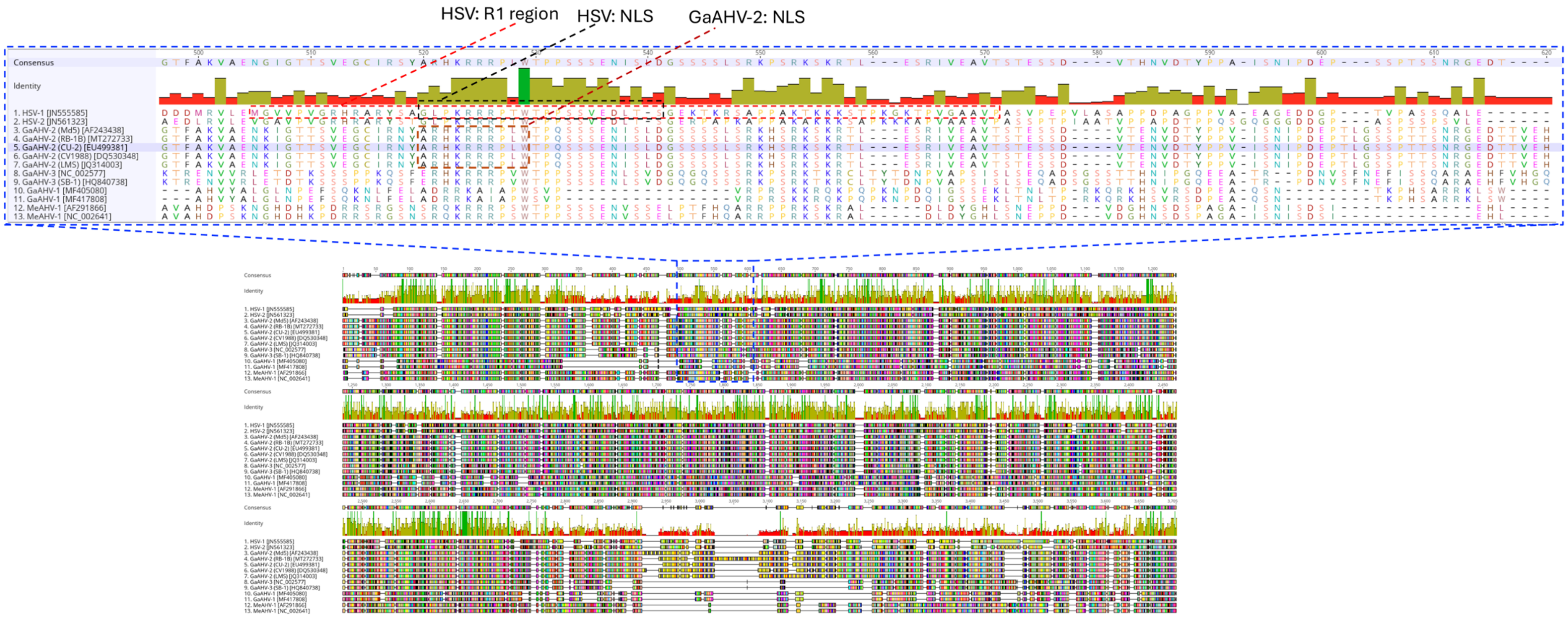

Fig. S1: Comparative analysis of the UL36 R1 region and nuclear localisation signal (NLS) across selected herpesviruses. Comparative visualisation of UL36 amino acid sequences from selected herpesviruses generated using Geneious. The R1 region encoding the NLS of HSV-1 (residues 375–455) and HSV-2 (Hennig et al., 2014) is compared with and aligned to the corresponding region in GaAHV2, with highlighted regions indicating conserved and divergent motifs.

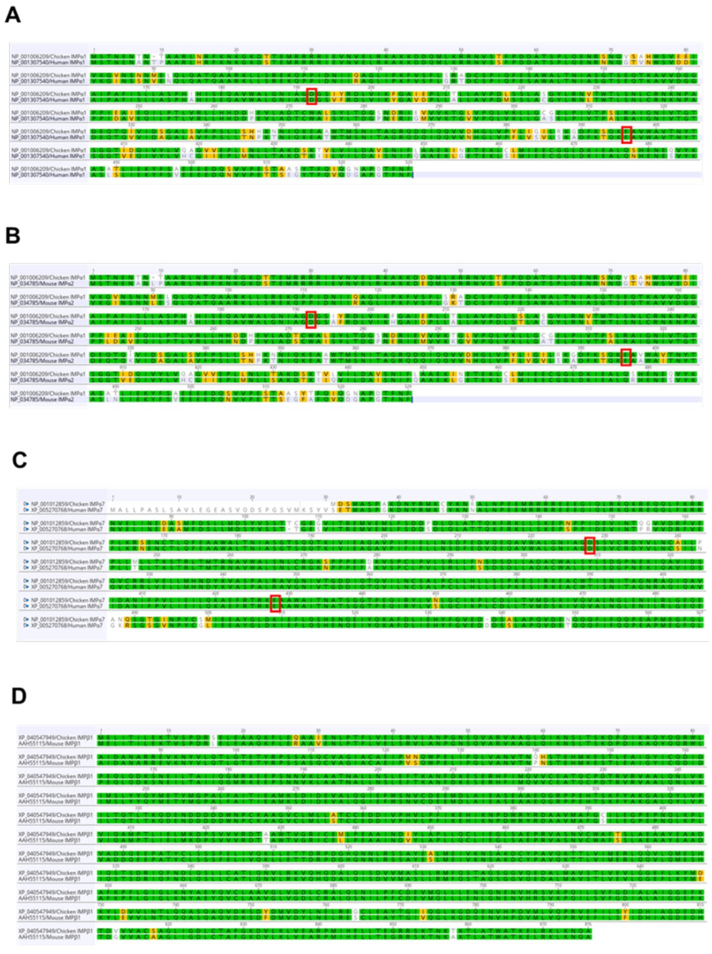

Fig. S2: Alignment of human/mouse and chicken importin amino acid sequences. (a) Human IMPα1 and chicken IMPα1 (83.36% homology) (b) Mouse IMPα2 and chicken IMPα1 (82.23% homology) (c) Human IMPα7 and chicken IMPα7 (93.85% homology) (d) Mouse IMPβ1 and chicken IMPβ1 (97.37% homology). Alignment was performed using the MAFFT L-INS-I algorithm within Geneious Prime (version 7.388). Conserved major (P2) and minor (P2′) binding sites of human/mouse and chicken IMPα are highlighted in the red box within the alignment.

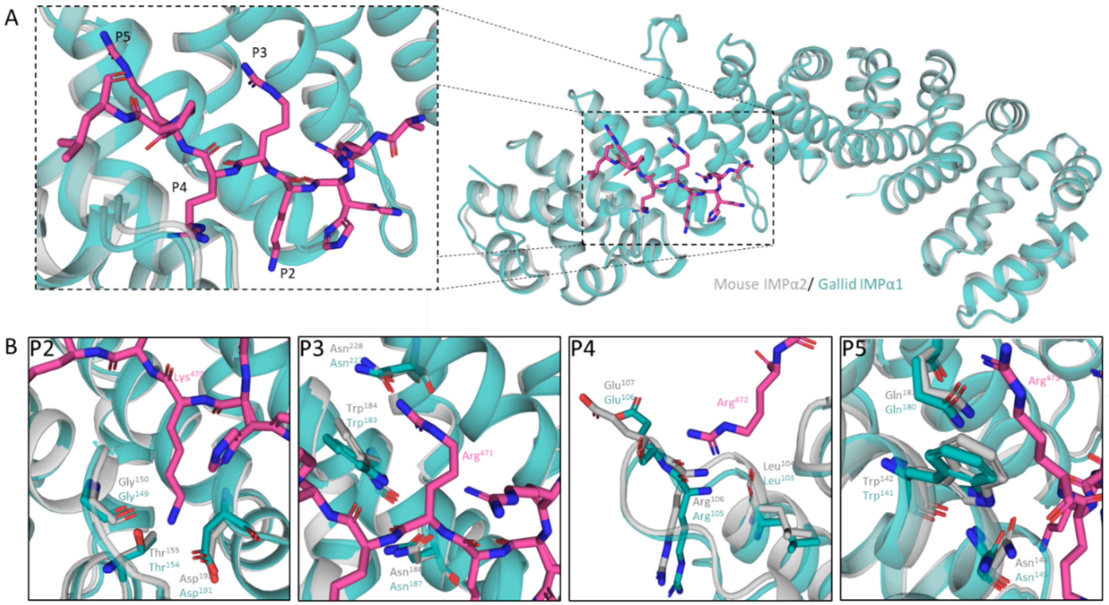

Fig. S3: Conservation of the IMPα major binding site between mouse IMPα1 (named as IMPα2 in our study) and chichen IMPα1. Residues 70-528 of chicken (*Gallus gallus*) IMPα1 (GenBank accession no. NP_001006209.2) were modelled using AlphaFold 3 (494-528 hidden in figure). **A.** Structural superposition of mouse IMPα2 (grey cartoon) and chicken IMPα1 (teal cartoon), highlighting the with the GaAHV2 UL36 NLS as pink sticks. **B.** Close-up view of the NLS-binding site showing key interactions observed in the mouse IMPα2 crystal structure. Interacting residues are depicted as sticks, illustrating strict conservation at the binding interface.

Table S1: Amino acid sequence identities among selected herpesvirus UL36 genes

	HSV-1 [JN555585]	HSV-2 [JN561323]	GaAHV-2 (Md5) [AF243438]	GaAHV-2 (RB-1B) [MT272733]	GaAHV-2 (CU-2) [EU499381]	GaAHV-2 (CV1988) [DQ530348]	GaAHV-2 (LMS) [JQ314003]	GaAHV-3 [NC_002577]	GaAHV-3 (SB-1) [HQ840738]	GaAHV-1 [MF405080]	GaAHV-1 [MF417808]	MeAHV-1 [AF291866]	MeAHV-1 [NC_002641]
HSV-1 [JN555585]			23.34	23.005	23.373	23.512	23.37	24.385	24.385	17.535	17.505	23.957	23.957
HSV-2 [JN561323]	77.669		23.436	23.162	23.535	23.587	23.531	24.368	24.332	17.425	17.395	24.051	24.051
GaAHV-2 (Md5) [AF243438]	23.34	23.436		96.867	98.776	98.238	98.718	50.339	50.516	17.089	17.089	47.282	47.282
GaAHV-2 (RB-1B) [MT272733]	23.005	23.162	96.867		97.582	96.501	97.557	49.708	49.883	16.889	16.889	46.647	46.647
GaAHV-2 (CU-2) [EU499381]	23.373	23.535	98.776	97.582		98.292	99.55	50.698	50.877	17.212	17.212	47.588	47.588
GaAHV-2 (CV1988) [DQ530348]	23.512	23.587	98.238	96.501	98.292		98.235	50.668	50.846	17.211	17.211	47.532	47.532
GaAHV-2 (LMS) [JQ314003]	23.37	23.531	98.718	97.557	99.55	98.235		50.489	50.666	17.153	17.153	47.39	47.39
GaAHV-3 [NC_002577]	24.385	24.368	50.339	49.708	50.698	50.668	50.489		99.12	17.446	17.446	47.408	47.408
GaAHV-3 (SB-1) [HQ840738]	24.385	24.332	50.516	49.883	50.877	50.846	50.666	99.12		17.476	17.476	47.455	47.455
GaAHV-1 [MF405080]	17.535	17.425	17.089	16.889	17.212	17.211	17.153	17.446	17.476		99.964	17.057	17.057
GaAHV-1 [MF417808]	17.505	17.395	17.089	16.889	17.212	17.211	17.153	17.446	17.476	99.964		17.057	17.057
MeAHV-1 [AF291866]	23.957	24.051	47.282	46.647	47.588	47.532	47.39	47.408	47.455	17.057	17.057		100
MeAHV-1 [NC_002641]	23.957	24.051	47.282	46.647	47.588	47.532	47.39	47.408	47.455	17.057	17.057	100	
